# Mortality in Tuberous Sclerosis Complex in the United Kingdom, 2016–2022

**DOI:** 10.1111/jir.13225

**Published:** 2025-03-10

**Authors:** Callum Richard Thomas Kidson, Ne‐Ron Loh, Yasir Ahmed Syed

**Affiliations:** ^1^ University of Bristol Medical School Bristol UK; ^2^ Royal United Hospitals Bath UK; ^3^ Neuroscience and Mental Health Innovation Institute Cardiff UK; ^4^ School of Bioscience Cardiff University Cardiff UK

**Keywords:** everolimus, mortality, renal disease, SUDEP, tuberous sclerosis complex

## Abstract

**Background:**

Tuberous sclerosis complex (TSC) is a genetic condition caused by mutations in either *TSC1* or *TSC2* genes, affecting around two million people globally. This study aims to examine causes of death in TSC and explore factors contributing to mortality in people with TSC in the United Kingdom in recent years following updated management and surveillance guidelines for the condition.

**Methods:**

Comprehensive analysis of the available medical records of the people seen at the largest lifespan TSC clinic in the United Kingdom who passed away between 2016 and 2022 was conducted. Disease‐related factors were identified, and the cause of death was determined. Where mortality cause was unobtainable, information was sought from the person's general practitioner, or their death certificate was obtained from the General Registry Office. Subsequently, the cohort was divided into subgroups to investigate potential risk factors for premature mortality. Our results were compared to that of previous TSC mortality studies.

**Results:**

The study consisted of 19 deaths. Nine deaths were unequivocally attributed to TSC. These fatalities were due to epilepsy (*n* = 3/19), aspiration pneumonia (*n* = 3/19), SEGA (*n* = 1/19), hepatic AML (*n* = 1/19) and pNET (*n* = 1/19). Other causes included malignant cancer (*n* = 6/19), sepsis (*n* = 2/19), COVID‐19 (*n* = 1/19) and stroke (*n* = 1/19). Renal failure was a secondary cause in two deaths.

**Discussion:**

Compared to limited previous mortality studies, this cohort appears to be less affected by SUDEP. This group is also more greatly affected by cancer and presents a potential link between early mortality and renal AML size. Moreover, a clearer role of intellectual disability in mortality of people with TSC may have been identified. Most causes of mortality in this TSC cohort are potentially prevented with suitable interventions earlier.

**Abbreviations**
CKD: chronic kidney disease
LAM: lymphangioleiomyomatosis
mTOR: mammalian target of rapamycin
PKD: polycystic kidney disease
pNET: pancreatic neuroendocrine tumour
SEGA: subependymal giant cell astrocytoma
SUDEP: sudden unexplained death in epilepsy
TSC: tuberous sclerosis complex

## 1 Background

Tuberous sclerosis complex (TSC) is an autosomal dominant genetic condition resulting from mutations in the *TSC1* or *TSC2* genes. These genes, located on chromosomes 9 and 16, encode hamartin and tuberin, respectively (Henske et al. 2016). Dysfunction of these proteins leads to hyperactivity of the crucial mammalian target of rapamycin (mTOR) growth pathway. Consequentially, hamartomas can arise in almost any organ resulting in various disease manifestations. Many of these tumours are treated effectively with mTOR inhibitors (Franz and Capal 2017). Notable hamartomas include renal angiomyolipomas (AML), subependymal giant cell astrocytomas (SEGA), cortical tubers and pulmonary lymphangioleiomyomatosis (LAM). Renal AMLs infiltrate viable renal tissue and can spontaneously haemorrhage, posing a potentially life‐threatening and tissue damaging risk (Peron et al. 2018). During childhood, SEGAs exhibit slow growth and typically cease growing by adulthood. In some cases, they may lead to obstructive hydrocephalus (Amin et al. 2013). LAM mainly impacts female sufferers, leading to recurrent pneumothoraces and gradual lung function decline, resulting in progressive breathlessness. Cortical tubers contribute to epilepsy development, commonly seen in individuals with TSC, along with intellectual disabilities (O'Callaghan et al. 2004). The disease also manifests through several other symptoms, including facial angiofibroma, ungual fibroma, retinal phakoma and cardiac rhabdomyoma, which typically resolve during childhood (Peron et al. 2018; Sciacca et al. 2014).

The Royal United Hospitals (RUH) in Bath hosts the UK's largest TSC lifespan clinic and receives referrals from hospitals in the region for review and treatment. A previous mortality study of the clinic population from 1981 to 2015 was contradicted by a recent large‐scale US mortality study of 18 TSC centres in the United States covering 2008 to 2020 (Parthasarathy et al. 2021; Amin et al. 2017). As many of the deaths covered by the UK study occurred, guidelines for TSC management have changed (Krueger et al. 2013). Typifying this is the example of everolimus, an mTOR inhibitor, which received health service funding in the United Kingdom in 2016 for treatment of renal AMLs and SEGAs and for managing TSC‐associated epilepsy in 2018 (Specialised Commissioning Team 2016a, 2016b, 2018). In early 2017, the first person at the Bath clinic initiated everolimus therapy.

Although renal disease has long been acknowledged as a major factor in mortality, the US study revealed no deaths directly linked to it, but a similar proportion attributed to sudden unexplained death in epilepsy (SUDEP) as previously reported (Parthasarathy et al. 2021; Amin et al. 2017; Shepherd et al. 1991). This may be in part influenced by changed management and surveillance guidelines, though it is difficult to know as limited literature exists outside the United Kingdom on changing mortality patterns.

The objective of this study is to investigate mortality causes among individuals with TSC in the United Kingdom given the substantial changes to treatment of the condition. Additionally, it aims to compare these findings with data from previously published studies, seeking to establish correlations and divergences.

## 2 Methods

### 2.1 Ethics

This work was registered as a service audit (ID 4096), as such ethical approval and familial consent were not necessary at the RUH.

### 2.2 Data Collection and Stratification

A thorough review of available clinical notes for people seen at the Bath clinic from 2016 to 2022 was performed; this identified those who had died and allowed collection of demographic details of patients at the clinic, namely, age and sex. Causes of death were determined using hospital records, general practitioner records and death certificates from the General Registry Office when required.

Data on TSC‐related comorbidities of these people were collected to group and analyse them, aiming to identify potential disease‐related risk factors for premature death (see Table 1). People with intellectual disabilities were categorised as having mild, moderate or severe intellectual disability per the Department of Health and Social Care's definitions (Department of Health and Social Care 2001). If causes directly linked to TSC were identified, they were classified as TSC‐related deaths; if TSC involvement could not be ruled out, they were considered possibly TSC‐related deaths; and deaths without evidence of TSC involvement were labelled as unrelated to TSC. Rare conditions that are strongly linked to TSC were identified using the Tuberous Sclerosis Registry to Increase Disease Awareness (TOSCA) (Sauter et al. 2021).

**TABLE 1:** TSC‐related comorbidities.

Comorbidity	*n*	*N* ^a^	%
Intellectual disabilities—any	15	19	79%
Intellectual disabilities—mild	2	19	11%
Intellectual disabilities—severe	13	19	68%
Epilepsy	17	19	89%
SEN or SEGA^a^	12	14	86%
Hepatic angiomyolipoma^a^	2	10	20%
Renal angiomyolipoma	15	19	79%
Lymphangioleiomyomatosis	4	16	25%
Facial angiofibroma	16	19	84%
Hypermelanic macule^a^	1	9	11%
Hypomelanic macule^a^	10	13	77%
Retinal lesion^a^	1	14	7%
Ungual fibroma^a^	7	14	50%
Shagreen patch^a^	4	9	44%
Cephalic plaque^a^	1	8	13%
Cardiac rhabdomyoma^a^	6	9	67%

Abbreviations: SEGA = subependymal giant cell astrocytoma, SEN‐subependymal nodule.

^a^ Data were not available for everyone.

### 2.3 Statistical Analysis

Although results from individual causes were envisaged as being descriptive, Fisher's exact test was used to compare prevalence of causes previously reported to be high prevalence. Reported deaths in the literature with unknown causes were excluded from comparison. Fisher's exact test was also used to compare proportions of TSC‐related and possibly TSC‐related deaths with data from other published cohorts. The age at death for each potential disease‐related risk factor was compared using independent *t*‐tests. In cases where data on tumour size were sufficient, regression analysis was conducted.

## 3 Results

### 3.1 Cohort Demographics

From 2016 to 2022, 369 people sought care at the Bath clinic. Of these, 183 (49.6%) were female, whereas 186 (50.4%) were male. These patients were aged between 0 and 78 years with a median age of 27 years (interquartile range [IQR] 15.3–40.0) and a mean age of 29 years (standard deviation [SD] 16.7).

In this period, a total of 19 deaths were identified. Of these, 8 were female, and 11 were male. Among these individuals, 79% (15 deaths) were found to have intellectual disabilities, with 68% (13 deaths) having severe intellectual disabilities. The median age of death was 41 years (IQR 27.5–56.5) with a mean of 42 years (SD 15.9) and a range of 21–73 years. Those that died were statistically significantly older than the cohort age (*p* < 0.001). People with intellectual disabilities had a younger median age of death at 40 years (range of 21–73, mean 39.7), whereas those without such disabilities had a median age of 56.5 years (range of 27–67, mean 51.75) (*p* = 0.251).

Median follow‐up for these people was 14 years (IQR 6.9–20.9). Six people had a follow‐up period greater than 20 years, and another six had a follow‐up period ranging from 10 to 20 years. Seven people were followed up for less than 10 years. Unfortunately, one person passed away before any follow‐up appointments took place. There was no significant difference in the age of death between men (median 41 years) and women (median 40.5 years) (*p* = 0.984). Total reported deaths had a similar distribution between sexes, aligning with findings from previous mortality studies (Shepherd et al. 1991; Amin et al. 2017; Parthasarathy et al. 2021).

Two people who passed away were undergoing treatment with sirolimus, an everolimus analogue, whereas one person had received everolimus intermittently. Sirolimus was indicated for immunosuppression for a renal transplant in both cases, and the everolimus was indicated for SEGA and renal AMLs.

According to medical records, genetic testing was done for 5 people out of the 19 who passed away. Two of them had no mutations, but one with hepatic AML had *TSC2* mutations. Two people had *TSC2* mutations and one had *TSC1* mutations. Additionally, 2 people had polycystic kidney disease (PKD), possibly linked to *TSC2*/*PKD1* contiguous deletion syndrome (Velasco et al. 2013).

### 3.2 Causes of Death

Nine people had a cause of death that was TSC‐related. Of these nine, two individuals passed away due to aspiration pneumonia likely related to seizure, but another's cause of death was recorded as respiratory failure, although their recent medical history suggests it was also aspiration pneumonia. These three all had severe intellectual disabilities. One person likely died from SUDEP, but no post‐mortem was available to exclude other causes. Another individual entered status epilepticus secondary to sepsis and passed away. Additionally, one person had a seizure resulting in a fatal traumatic haemorrhage. Another person's cause of death was obstructive hydrocephalus caused by a SEGA, whereas another succumbed to a metastatic pancreatic neuroendocrine tumour (pNET). Lastly, one person's cause of death was obstruction of the inferior vena cava by a hepatic AML.

**FIGURE 1:**
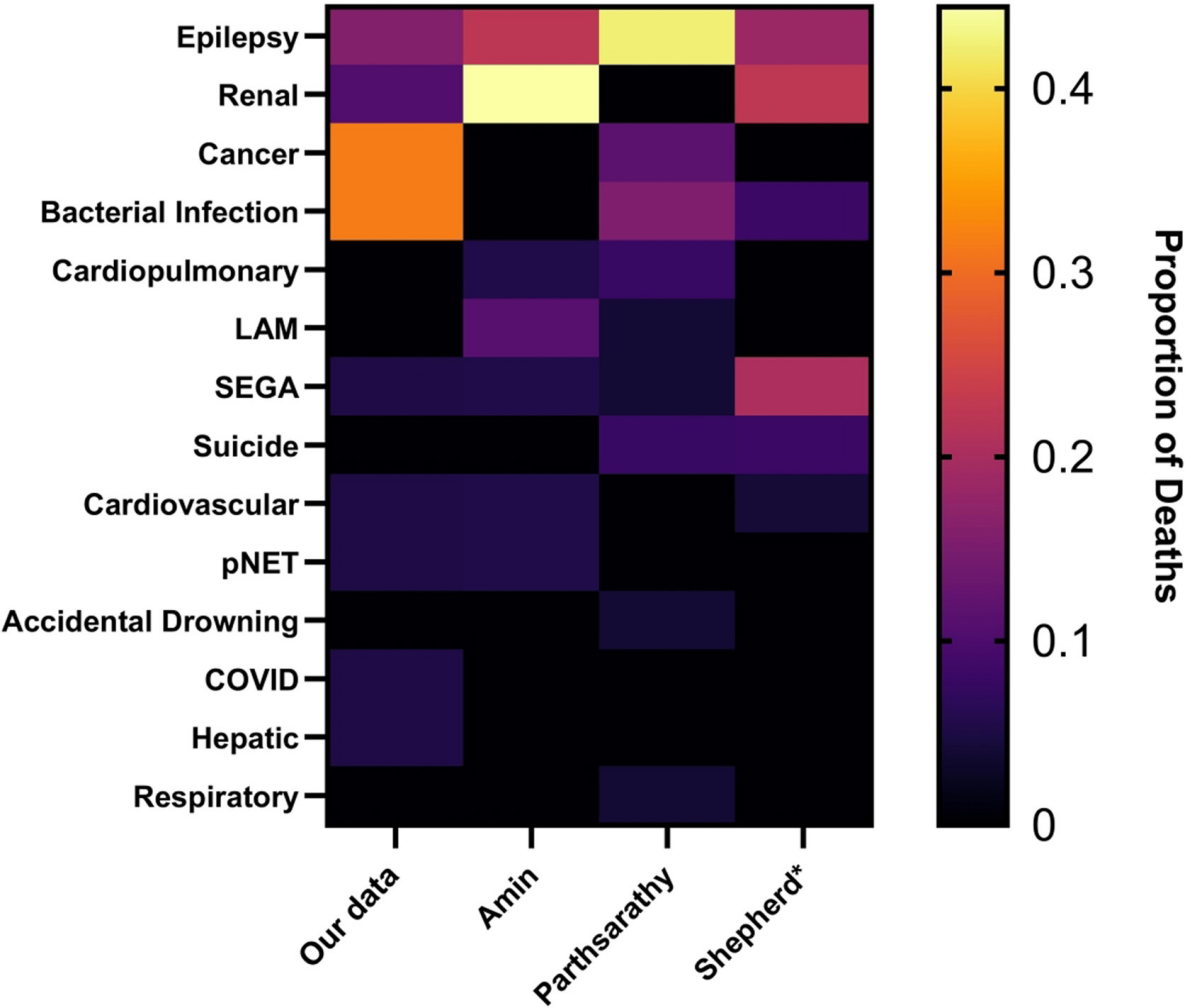
Heatmap of causes of death in published mortality cohorts compared to our data. *Data from Shepherd et al. (1991) does not include non‐TSC‐related causes. Graph shows the proportion of deaths in each cohort caused by listed grouped causes with heat scale seen on the right‐hand side of the figure.

**FIGURE 2:**
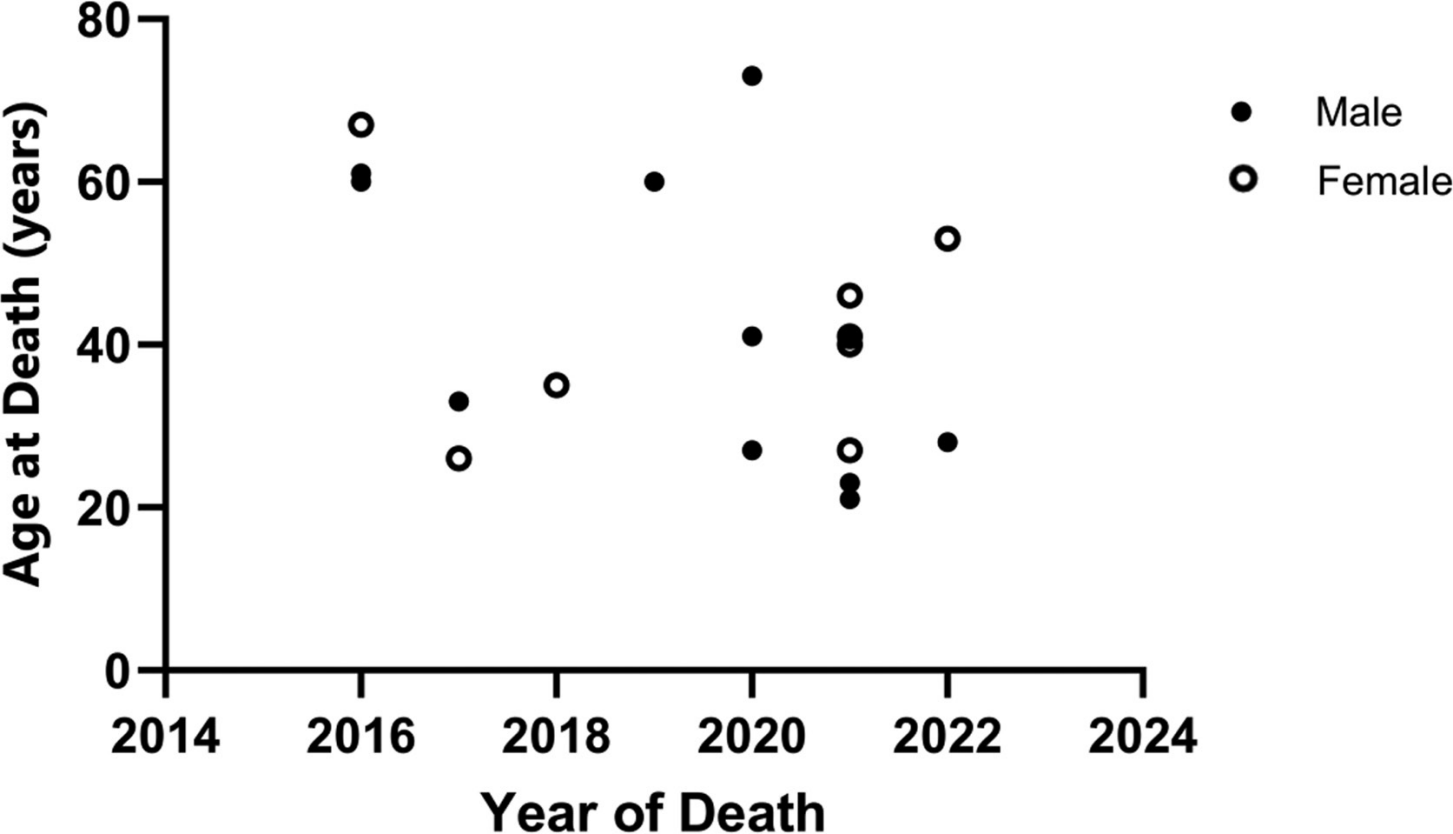
Scatter plot of the year of each death and the age of death in that year. Everyone from this mortality cohort is represented. The figure demonstrates which points represent men and which represent women.

For three people, the cause of death was possibly TSC‐related. One individual's cause of death was sepsis discovered post‐mortem, whereas another passed away from sepsis and renal failure (see Figures 1 and 2). On this death certificate, renal failure was a secondary cause of death and not listed as secondary to sepsis. This was likely due to the comorbid CKD, meaning the involvement of TSC in this death could not be ruled out. The final death similarly was attributed primarily to a COVID‐19 respiratory infection and secondarily to renal failure. This person had CKD due to PKD, and thus, the involvement of TSC could also not be ruled out.

Among the causes not normally known to be associated with TSC and therefore deemed unrelated, the most common were malignant cancers causing five deaths and probably a sixth where the person had recurrent sigmoid volvuli at an age where this would be suspicious of cancer; however, there was no post‐mortem to confirm this. Two deaths were due to gastrointestinal malignancies, but a non‐TSC temporal brain tumour, meningioma, melanoma and lymphoma each claimed a life. None of these malignancies have been found to be of greater prevalence in TSC (Sauter et al. 2021). Only one of these people did not have an intellectual disability. There was one death due to a stroke.

### 3.3 TSC Comorbidities

When comparing the age of groups with and without individual comorbidities related to TSC (see Table 2), it was observed that people with epilepsy had a shorter lifespan than those without (*p* = 0.012). No other significant differences were found. Additionally, there were no statistically significant differences in age at death for deaths related, possibly related, or unrelated to TSC.

**TABLE 2:** Comparison of median ages among subgroups.

Risk factor	People with factor	Median age	People without factor	Median age	*p*
Comorbidities					
Cardiac rhabdomyoma	6	26.5	3	35.0	0.167
Epilepsy	17	40.0	2	70.0	0.012
Facial angiofibroma	16	40.5	3	60.0	0.265
Hepatic angiomyolipoma	2	33.0	8	41.0	0.968
Intellectual disabilities	15	40.0	4	56.5	0.251
Lymphangioleiomyomatosis	4	43.5	12	37.0	0.535
Renal angiomyolipoma	15	41.0	4	25.5	0.095
Shagreen patches	4	27.0	5	54.0	0.200
Ungual fibroma	7	46.0	7	40.0	0.927
Demographics					
Residential care home	8	41.0	11	27.5	0.098
Sex	11 (M)	41.0	8 (F)	40.5	0.984
Cause of death					
TSC‐related	9	41.0	10	40.5	0.589
Excluding possibly related in comparison group	9	41.0	7	40.0	0.656
TSC‐related or possibly TSC‐related	12	41.0	7	40.0	0.602
Other					
Age of our cohort compared with Amin et al.'s cohort	18 (*Amin*)	33.0	19 (*our data*)	41.0	0.395
People receiving mTOR inhibition	3	43.0	16	40.0	0.718

**FIGURE 3:**
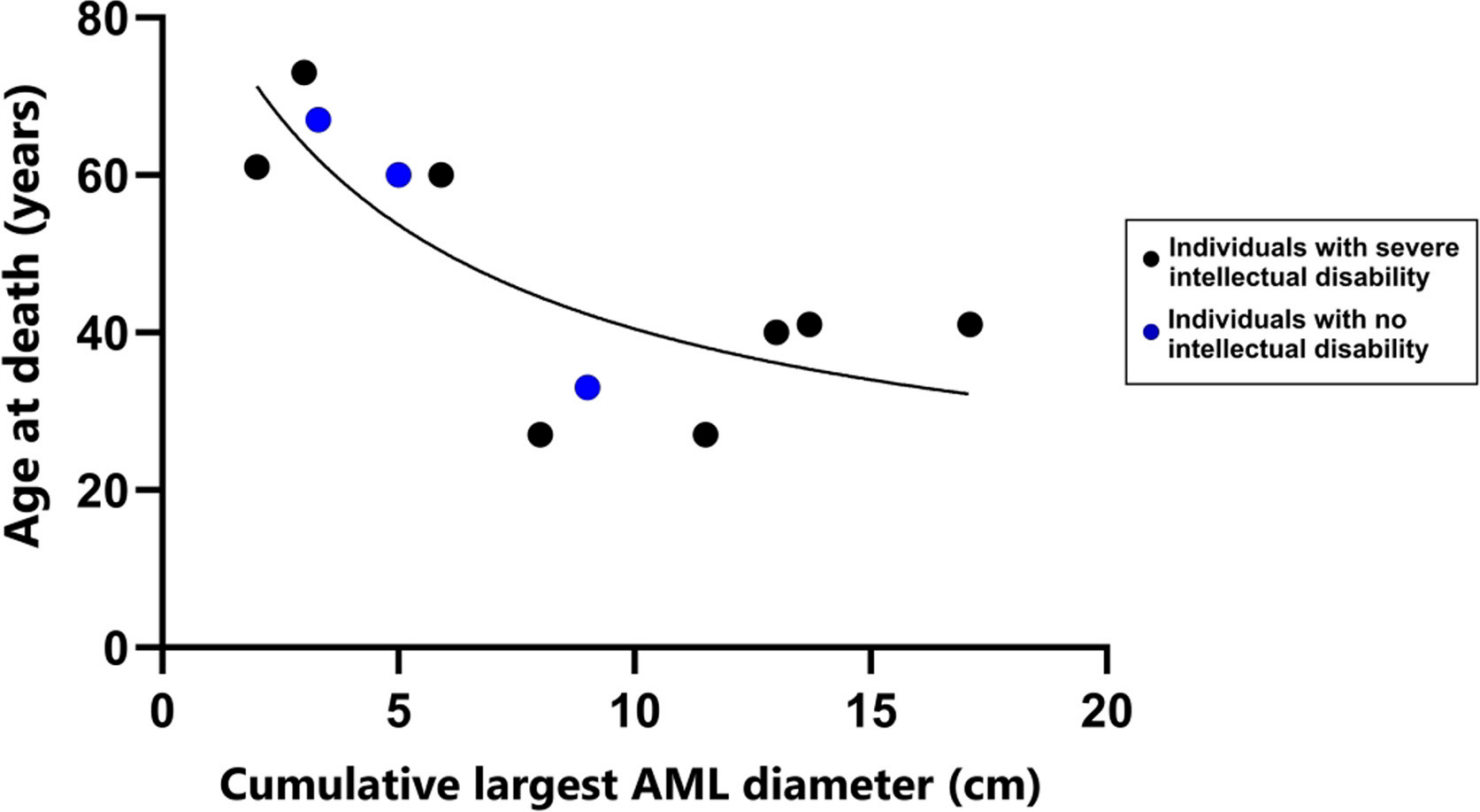
Scatter plot showing the correlation between burden of TSC renal disease and age of death. People for which these data were available were plotted onto a simple scatter plot comparing their age of death with the burden of their renal disease. The figure demonstrates whether or not these individuals had an intellectual disability. The best fit line is that used to calculate the best non‐linear regression value (*R* ^2^) of 0.636.

Furthermore, when examining the cumulative diameter of the largest renal AMLs from all native kidneys prior to any embolisation and correlating it with the age of death for the 11 people for whom complete data were available, a significant negative correlation was discovered using simple linear regression (*p* = 0.014), with an *R* ^2^ value of 0.509. The best fitting non‐linear model achieved an *R* ^2^ value of 0.636 (see Figure 3).

## 4 Discussion

Our data could suggest, in line with established evidence, that intellectual disabilities may have impact life expectancy in those with TSC when compared to the general population (University of Bristol 2021). Although SUDEP occurrence possibly appears to have decreased when compared to recent literature, renal disease burden remains correlated with mortality in this cohort, as does epilepsy (Parthasarathy et al. 2021; Amin et al. 2017). The COVID‐19 pandemic likely affected findings, with many in the group dying from cancers, potentially due to delayed care. Data show a higher proportion of deaths from causes related to TSC in the United States compared to the United Kingdom. The comparatively young age of the overall clinic population compared to this mortality cohort could suggests TSC may carry a true life expectancy greater than that described by these data.

No deaths reported in the period of 2016–2022 were attributed to renal AML bleeds where previously 3 of 18 deaths in Amin et al. reported this as the cause. Despite this, the correlation found between renal AML size and age at death suggests that management of renal disease remains important for prognosis. Evidence already exists that presence of renal disease in TSC correlates with an earlier death (Peng et al. 2021), in line with evidence that comorbid renal disease is a mortality predictor and in line with current guidance (Hansrivijit et al. 2021; Northrup et al. 2021). Renal morbidity was significant in this cohort: Two people had renal transplantation, one of whom had no native kidneys; two others underwent nephrectomies due to bleeding; and one person was embolised for bleeding AML. CKD burden was also high due to the prevalence of AMLs with 15 people reported to have them. Of note is the most recent TSC mortality publication that reports no death related to renal involvement (Parthasarathy et al. 2021), whereas this cohort reports two people dying with renal failure as a secondary cause of death.

In the qualifying period, only one of the 19 deaths was due to SUDEP, a lower proportion than previously reported (Parthasarathy et al. 2021; Amin et al. 2017). This is possibly influenced by work to increase SUDEP prevention in the United Kingdom since the previous publication (Shankar et al. 2017) or the increase in proportion of other mortality causes. Both this study and the previous Bath study report lower proportions of SUDEP deaths in the United Kingdom compared to the United States (Parthasarathy et al. 2021). The difference in SUDEP prevalence in this cohort compared to the most recent US study is statistically significant (*p* = 0.007), but this is not the case when compared with the previous Bath study (*p* = 0.180). As SUDEP risk is known to be reduced by effective epilepsy control (Maguire et al. 2015), an explanation of this difference could relate to the United Kingdom having universal healthcare available through the National Health Service (NHS). This means people are not subject to varying standards of care by virtue of their insurance status as they are in many countries. However, when all deaths related to epilepsy in this cohort are compared with this US cohort, the difference does not maintain significance (*p* = 0.102). Comparison with earlier studies is difficult as all deaths related to epilepsy in Shepherd et al. are reported as status epilepticus. This may include SUDEP as historically these have been poorly differentiated (Nashef et al. 2012); the authors also acknowledged that these causes were not verified for their study.

In this mortality cohort, four people had LAM, but no deaths were attributed to it. This contrasts with previous studies (Amin et al. 2017; Parthasarathy et al. 2021; Shepherd et al. 1991). The low prevalence of LAM within this cohort may be one explanation for this (see Table 1). Records for the two LAM deaths reported by Amin et al. were locatable. They occurred before the only national LAM centre in the United Kingdom opened at which many people from the Bath clinic are now seen for their LAM. With better monitoring, it is possible that people with LAM are achieving better outcomes. Moreover, these deaths occurred before the first trials showing effective use of mTOR inhibition in treating LAM (Bissler et al. 2008). Everolimus may also play some role here given that if it were prescribed for comorbid renal AMLs or SEGA, it may prevent development of clinically significant LAM.

Though only one death was attributed to SEGA, morbidity within this cohort remains high. Four people had SEGA resections during their lifetime, and only two of 14 people for whom these data were available did not have SEGAs or subependymal nodules.

This mortality cohort had nine TSC‐related deaths (47%) and two possibly TSC‐related deaths (11%). Proportionally less deaths were related or possibly related to TSC than in the Parthasarathy et al. cohort (*p* = 0.006). This difference was not significant when compared with Amin et al. (*p* = 0.125) or Shepherd et al. (*p* = 0.123). Shepherd did not report causes of deaths unrelated to TSC, so verification of their relatedness with contemporary knowledge is impossible.

Though comparison with Amin et al. failed to reach statistical significance, there were proportionally fewer deaths TSC‐related or possibly TSC‐related. An explanation for this, besides statistical variation, could be the expansion of mTOR inhibition use for TSC from eight people in the clinic at the end of 2015 to 67 now. There is also a shift to preventative treatment rather than waiting for symptoms. Nobody in the previous Bath mortality group received mTOR inhibition. In this mortality cohort, three people received mTOR inhibition, two were receiving sirolimus for their kidney transplants, and the people who died from their SEGA had intermittently taken everolimus for their SEGA and renal AMLs but stopped due to tolerability issues.

Further explanation could be provided by potential influence from the COVID pandemic and its impact on care pathways in the United Kingdom. Eleven (58%) of the 2016–2022 deaths occurred following the start of the COVID pandemic, defined as the date of the first lockdown restrictions in the United Kingdom. This warrants caution in interpreting results due to the probable influence of the COVID pandemic on causes of deaths, specifically cancer deaths. Perhaps, the death rate might have been much lower in the absence of the pandemic.

Of the non‐TSC‐related cancers, two thirds of them occurred after the pandemic started when there was extraordinary pressure on cancer diagnosis pathways in the UK delaying treatment. Moreover, fewer people than expected were diagnosed with cancer during this period suggesting reduced detection, which leads to poorer outcomes (Morris et al. 2021).

This affected people with and without intellectual disabilities; the person that died from a meningioma was in their forties and had severe intellectual disabilities so required a general anaesthetic for an MR,I which, due to backlogs, was delayed for a year. The person that died from lymphoma was in their twenties with no intellectual disabilities. Their diagnosis was delayed due to a wrong diagnosis of long COVID, which proved fatal due to the lymphoma's high grade. From the TOSCA database, it was observed that beyond renal cell carcinoma and thyroid cancer, malignancies were surprisingly uncommon in TSC, given the genetic basis of the condition (Sauter et al. 2021; Henske et al. 2016).

Alternatively, this difference could be explained by the older age of this mortality cohort compared to previous ones. Our cohort had an older age range (median 41, IQR 27.5–56.5) compared with Amin et al. (median 33, IQR 26–46) and an older median age than Parthasarathy et al. (28 years), though the difference with the Amin cohort is not statistically significant (*p* = 0.395). Despite the clinic seeing both adult and paediatric ages, we had no reported deaths younger than 21 years in contrast to Parthasarathy et al. reporting 41.9% of deaths occurring below this age.

People are referred to the clinic for support in managing their condition by coordinating care between providers. Therefore, our data may be insensitive to some causes such as potential neonatal deaths from causes such as cardiac rhabdomyoma that later regress. This could explain why there are no deaths in this age group. Alternatively, explanation could be drawn from the differing healthcare systems.

If treating this mortality cohort and the previous Bath cohort as one, differences between UK and US populations are more confidently identified. This group covers the period 1981–2022 inclusive compared to Parthasarathy et al. covering 2008 to 2020. Thirty‐seven deaths were reported in this period with no unknown causes. Parthasarathy reported 33 deaths, of which seven had unknown causes. Proportionally more people in the US cohort died of causes TSC‐related or possibly TSC‐related than in the UK cohort (*p* = 0.037). Proportionally more deaths in the US cohort were attributable to SUDEP (*p* = 0.017), and proportionally more deaths in the UK cohort were attributable, including as a secondary cause, to renal causes (*p* = 0.004). Inclusion of secondary causes here is valuable as it highlights that potential metabolic abnormalities from renal insufficiency may lead to worsened prognosis. These stark differences further suggest that these distinct populations have variable TSC healthcare challenges with evidence of a higher burden, at least on mortality, of renal disease in the United Kingdom. When comparing all deaths with epilepsy involved, the SUDEP differences are no longer statistically significant (*p* = 0.053), and comparison of the UK cohort with Shepherd et al. suggests there is no difference in mortality burden of epilepsy (*p* > 0.999).

With only four people without intellectual disabilities in this mortality cohort, it is possible that the early median age of death (41 years) is influenced by previously proven healthcare disparities between those with and those without intellectual disability (University of Bristol 2021). Of the people without intellectual disabilities that died, three died from rapid onset causes and one of a metastatic pNET. The two people with mild intellectual disabilities died in their 20s of acute causes: melanoma and SUDEP.

Another factor that may bear influence on this young age of death is that people with TSC are likely to develop malignancies at a younger age. However, available evidence suggests this is the case for only some malignancies, despite the mTOR pathway's clear role in regulation of cell growth (Sauter et al. 2021; Henske et al. 2016).

It is difficult to do more than hypothesise about the reasons for difference between the populations compared in this paper given the variability in geography as well as time periods. However, it would be reasonable to suggest they are complex and multifactorial. The differing healthcare availability between locations is itself one factor, along with differing global events over the varying periods. Greater awareness of TSC and published guidance is another factor resulting in increased surveillance possibly influencing mortality and morbidity (Krueger et al. 2013). Furthermore, increased availability of mTOR inhibition as treatment for the mortality causes that have previously shown increased prevalence may be an influencing factor.

This study significantly departs from previous literature by categorising aspiration pneumonia as a TSC‐related cause of death: Everyone in this cohort with intellectual disabilities is presumed to have them because of their TSC; all those in this cohort that died of aspiration pneumonias had severe intellectual disabilities; aspiration pneumonias represent a disproportionate number of deaths in those with intellectual disabilities (University of Bristol 2021); it is therefore appropriate to classify this cause as TSC‐related. The authors believe that this rationale aligns more closely with the World Health Organisation's guidelines for reporting deaths.

Genetic testing is not routine in the United Kingdom for those with intellectual disabilities that limit the likelihood of them going on to reproduce. As these people also receive regular disease monitoring, a clinical diagnosis is sufficient as an indication for treatment (Northrup et al. 2021). Any testing is not performed at the clinic, as such obtaining these data was labour intensive and not possible for individuals in the wider cohort who had not died.

One strength of the study, compared to previous ones, is the use of secondary causes of death to highlight renal disease as an ongoing contributor to mortality.

### 4.1 Limitations

The Bath clinic primarily receives referrals from secondary and tertiary care providers, which may create a bias towards people with severe TSC. It is a supra‐regional specialist centre and therefore does not function in the same way as conventional clinics making thorough evaluation of a person's whole clinical history difficult. Its functioning may also increase predilection to older people as infant mortalities may not be referred to the centre before death. Furthermore, identifying how much overlap is present in the whole clinic cohort now compared with the 2016 data (Amin et al. 2017) is not feasible given a transition at RUH to digital note systems. However, it should be noted that the clinic has expanded its capacity since the publication by Amin et al.

Fortunately, the size of this mortality cohort is small; however, this somewhat limits meaningful interpretation of data especially in comparison to other larger studies. As such, much of the analysis is descriptive. The limited availability of literature for comparison further impacts comparisons given that the previous Bath paper covers a much longer timeframe than the multicentre US paper. This papers also study populations subject to differing healthcare systems.

## 5 Conclusions

These data further demonstrate what is already known about the importance of monitoring renal disease in people with TSC. Not only are renal disease manifestations potentially contributing to mortality in two cases, but the size of the renal hamartomas also appears to correlate with an earlier age of death. This warrants further investigation given the apparent unrelatedness of renal morbidity to many of the causes of death.

Many deaths were attributed to cancer. This may have been exacerbated by both the COVID pandemic and disparities in outcomes for those with intellectual disabilities and therefore a reduced ability to present their symptoms. Regardless, this may highlight a need to add cancer prevention into the TSC expert's extensive considerations when consulting with this group.

Intellectual disability may also have a further covert role in mortality in those with TSC given the high prevalence of infection in this group from causes that are known to be of greater prevalence in those with intellectual disabilities. Moreover, effective epilepsy management plays a vital role in reducing SUDEP burden and other epilepsy‐related deaths.

An absence of deaths reported due to LAM may indicate the effectiveness of centralised specialist knowledge in this area as well as potentially being the result of more widespread mTOR inhibition therapy. Overall, it appears that most deaths have the potential to be prevented with many of them having a modifiable disease course. Additionally, the average age of the clinic population being greater than that of the mortality cohort may suggest the life expectancy suggested by these data is younger than true life expectancy for those with TSC.

## Conflicts of Interest

The authors declare no conflicts of interest.

## Data Availability

The data that support the findings of this study are available on request from the corresponding author. The data are not publicly available due to privacy or ethical restrictions.

## References

[jir13225-bib-0001] Amin, S. , M. Carter , R. J. Edwards , et al. 2013. “The Outcome of Surgical Management of Subependymal Giant Cell Astrocytoma in Tuberous Sclerosis Complex.” European Journal of Paediatric Neurology 17: 36–44. 23183057 10.1016/j.ejpn.2012.10.005

[jir13225-bib-0002] Amin, S. , A. Lux , N. Calder , M. Laugharn , J. Osborne , and F. O'Callaghan . 2017. “Causes of Mortality in Individuals With Tuberous Sclerosis Complex.” Developmental Medicine and Child Neurology 59: 612–617. 27935023 10.1111/dmcn.13352

[jir13225-bib-0003] Bissler, J. J. , F. X. McCormack , L. R. Young , et al. 2008. “Sirolimus for Angiomyolipoma in Tuberous Sclerosis Complex or Lymphangioleiomyomatosis.” New England Journal of Medicine 358: 140–151. 18184959 10.1056/NEJMoa063564 PMC3398441

[jir13225-bib-0004] Department of Health and Social Care . 2001. “Valuing People: A New Strategy for Learning Disability for the 21st Century.” Department of Health, London.

[jir13225-bib-0005] Franz, D. N. , and J. K. Capal . 2017. “mTOR Inhibitors in the Pharmacologic Management of Tuberous Sclerosis Complex and Their Potential Role in Other Rare Neurodevelopmental Disorders.” Orphanet Journal of Rare Diseases 12: 1–9. 28288694 10.1186/s13023-017-0596-2 PMC5348752

[jir13225-bib-0006] Hansrivijit, P. , Y. J. Chen , K. Lnu , et al. 2021. “Prediction of Mortality Among Patients With Chronic Kidney Disease: A Systematic Review.” World Journal of Nephrology 10: 59–75. 34430385 10.5527/wjn.v10.i4.59 PMC8353601

[jir13225-bib-0007] Henske, E. P. , S. Józwiak , C. J. Kingswood , J. R. Sampson , and E. A. Thiele . 2016. “Tuberous Sclerosis Complex.” Nature Reviews Disease Primers 2: 16035. 10.1038/nrdp.2016.35 27226234

[jir13225-bib-0008] Krueger, D. A. , H. Northrup , and The International Tuberous Sclerosis Complex Consensus Group . 2013. “Tuberous Sclerosis Complex Surveillance and Management: Recommendations of the 2012 International Tuberous Sclerosis Complex Consensus Conference.” Pediatric Neurology 49: 255–265. 24053983 10.1016/j.pediatrneurol.2013.08.002 PMC4058297

[jir13225-bib-0009] Maguire, M. J. , C. F. Jackson , A. G. Marson , and S. J. Nolan . 2015. “Treatments for the Prevention of Sudden Unexpected Death in Epilepsy (SUDEP).” Cochrane Databse of Systematic Reviews. 10.1002/14651858.CD011792.pub2 PMC6458047 27434597

[jir13225-bib-0010] Morris, E. J. A. , R. Goldacre , E. Spata , et al. 2021. “Impact of the COVID‐19 Pandemic on the Detection and Management of Colorectal Cancer in England: A Population‐Based Study.” Lancet Gastroenterology & Hepatology 6: 199–208. 33453763 10.1016/S2468-1253(21)00005-4 PMC7808901

[jir13225-bib-0011] Nashef, L. , E. L. So , P. Ryvlin , and T. Tomson . 2012. “Unifying the Definitions of Sudden Unexpected Death in Epilepsy.” Epilepsia 53: 227–233. 22191982 10.1111/j.1528-1167.2011.03358.x

[jir13225-bib-0012] Northrup, H. , M. E. Aronow , E. M. Bebin , et al. 2021. “Updated International Tuberous Sclerosis Complex Diagnostic Criteria and Surveillance and Management Recommendations.” Paediatric Neurology 123: 50–66. 10.1016/j.pediatrneurol.2021.07.011 34399110

[jir13225-bib-0013] O'Callaghan, F. J. K. , T. Harris , C. Joinson , et al. 2004. “The Relation of Infantile Spasms, Tubers, and Intelligence in Tuberous Sclerosis Complex.” Archives of Disease in Childhood 89: 530–533. 15155396 10.1136/adc.2003.026815 PMC1719953

[jir13225-bib-0014] Parthasarathy, S. , R. Mahalingam , J. Melchiorre , J. Harowitz , and O. Devinsky . 2021. “Mortality in Tuberous Sclerosis Complex.” Epilepsy and Behaviour 121: 108032. 10.1016/j.yebeh.2021.108032 34087679

[jir13225-bib-0015] Peng, J. H. , H. P. Tu , and C. H. Hong . 2021. “A Population‐Based Study to Estimate Survival and Standardized Mortality of Tuberous Sclerosis Complex (TSC) in Taiwan.” Orphanet Journal of Rare Diseases 16: 335. 34344419 10.1186/s13023-021-01974-3 PMC8330058

[jir13225-bib-0016] Peron, A. , M. P. Canevini , F. Ghelma , F. Di Marco , and A. Vignoli . 2018. “Healthcare Transition From Childhood to Adulthood in Tuberous Sclerosis Complex.” American Journal of Medical Genetics 178: 355–364. 30253036 10.1002/ajmg.c.31653 PMC6635672

[jir13225-bib-0017] Sauter, M. , E. Belousova , M. P. Benedik , et al. 2021. “Rare Manifestations and Malignancies in Tuberous Sclerosis Complex: Findings From the TuberOus SClerosis Registry to increAse Disease Awareness (TOSCA).” Orphanet Journal of Rare Diseases 16: 1–15. 34229737 10.1186/s13023-021-01917-y PMC8259106

[jir13225-bib-0018] Sciacca, P. , V. Giacchi , C. Mattia , et al. 2014. “Rhabdomyomas and Tuberous Sclerosis Complex: Our Experience in 33 Cases.” BMC Cardiovascular Disorders 14: 66. 24884933 10.1186/1471-2261-14-66 PMC4039990

[jir13225-bib-0019] Shankar, R. , E. J. Donner , B. McLean , L. Nashef , and T. Tomson . 2017. “Sudden Unexpected Death in Epilepsy (SUDEP): What Every Neurologist Should Know.” Epileptic Disorders 19: 1–9. 28218059 10.1684/epd.2017.0891

[jir13225-bib-0020] Shepherd, C. W. , M. R. Gomez , J. T. Lie , and C. S. Crowson . 1991. “Causes of Death in Patients With Tuberous Sclerosis.” Mayo Clinic Proceedings 66: 792–796. 1861550 10.1016/s0025-6196(12)61196-3

[jir13225-bib-0021] Specialised Commissioning Team . 2016a. “Clinical Commissioning Policy Statement: Everolimus (Votubia®) for Treatment of Angiomyolipomas Associated With Tuberous Sclerosis.” NHS England.

[jir13225-bib-0022] Specialised Commissioning Team . 2016b. “Everolimus for Subependymal Giant Cell Astrocytoma (SEGA) Associated With Tuberous Sclerosis Complex”.

[jir13225-bib-0023] Specialised Commissioning Team . 2018 “Clinical Commissioning Policy: Everolimus for Refractory Focal Onset Seizures Associated With Tuberous Sclerosis Complex (Aged 2 Years and Above).” NHS England.

[jir13225-bib-0024] University of Bristol . 2021. “The Learning Disabilities Mortality Review (LeDeR) Programme Annual Report.” Annual Report. University of Bristol.

[jir13225-bib-0025] Velasco, S. L. , A. C. Salas , C. V. Moreno , R. M. C. Rodriguez , F. J. M. Garcia , and R. S. de la Heras . 2013. “TSC2/PKD1 contiguous gene deletion syndrome.” Anales de Pediatría 79: 42–45. 23402778 10.1016/j.anpedi.2012.12.004

